# ﻿Ameripathidae, a new family of antipatharian corals (Cnidaria, Anthozoa, Hexacorallia, Antipatharia)

**DOI:** 10.3897/zookeys.1203.121411

**Published:** 2024-05-31

**Authors:** Jeremy Horowitz, Dennis M. Opresko, Santiago Herrera, Colleen M. Hansel, Andrea M. Quattrini

**Affiliations:** 1 Department of Invertebrate Zoology, National Museum of Natural History, Smithsonian Institution, Washington, DC, USA National Museum of Natural History, Smithsonian Institution Washington DC United States of America; 2 Department of Biological Sciences, Lehigh University, Lehigh, PA, USA Lehigh University Lehigh United States of America; 3 Department of Marine Chemistry and Geochemistry, Woods Hole Oceanographic Institution, Woods Hole, MA, USA Woods Hole Oceanographic Institution Woods Hole United States of America

**Keywords:** Deep sea, fauna of Puerto Rico, genome skimming, mesophotic, molecular phylogenetics, new genus, new species, taxonomy, ultraconserved elements

## Abstract

A new family of antipatharian corals, Ameripathidae (Cnidaria: Anthozoa: Antipatharia), is established for *Ameripathespseudomyriophylla* Opresko & Horowitz, **gen. et sp. nov.** The new family resembles Myriopathidae and Stylopathidae in terms of the morphology of the polyps and tentacles and the pinnulate branching of the corallum. Phylogenetic analysis using a genomic data set of 741 conserved element loci indicates that the new family is sister to a clade containing the Myriopathidae, Stylopathidae, Antipathidae, and Aphanipathidae.

## ﻿Introduction

Opresko (1972) re-examined Caribbean antipatharian corals originally described by L.F. de Pourtalès (1880). Among syntypes of *Antipathespicea* Pourtalès, 1880, Opresko found one specimen inconsistent with the species description, having only two rows of primary pinnules instead of four. Opresko provisionally identified this and two additional specimens from the Gulf of Mexico and Lesser Antilles as *Antipathesamericana* Duchassaing & Michelotti, 1860 (see Opresko 1972: 975–977); however, the type specimen of *A.americana* was not available for examination at that time. Later, a specimen labeled as a syntype of *A.americana* was discovered in the collections of the Regional Museum of Natural Sciences, Turin, Italy (Registration Number 78). This type specimen was considered to represent a valid species in the genus *Stylopathes* Opresko, 2006, in the family Stylopathidae Opresko, 2006 with a pinnulation pattern very different from the syntype of *A.picea* and the other two specimens provisionally assigned to *A.americana* by Opresko (1972). For example, they were unbranched but distinctly pinnulate with three orders of pinnules, with the primary pinnules in three to four rows along the length of the stem, and with secondary and tertiary pinnules in whorls with ≤ four members (see Opresko 2006: fig. 4a). It was concluded that the specimens originally referred to *A.americana* by Opresko (1972) represented a distinct and undescribed species; however, they could not be assigned to any nominal species known at the time. Although the pinnulation pattern of the species resembled that of species of the genus *Myriopathes* Opresko, 2001, the skeletal spines more closely resembled those of species of the family Stylopathidae.

Recently two samples were collected during an exploratory research cruise to the waters surrounding Puerto Rico conducted onboard the R/V Falkor (too) of the Schmidt Ocean Institute. These specimens matched the morphological characters of the specimens examined by Opresko (1972). We used an integrative approach, combining morphological and genomic data, to recognize these corals as representative of a new species, new genus, and new family.

## ﻿Materials and methods

### ﻿Specimen collection and deposition

**Figure 1.**
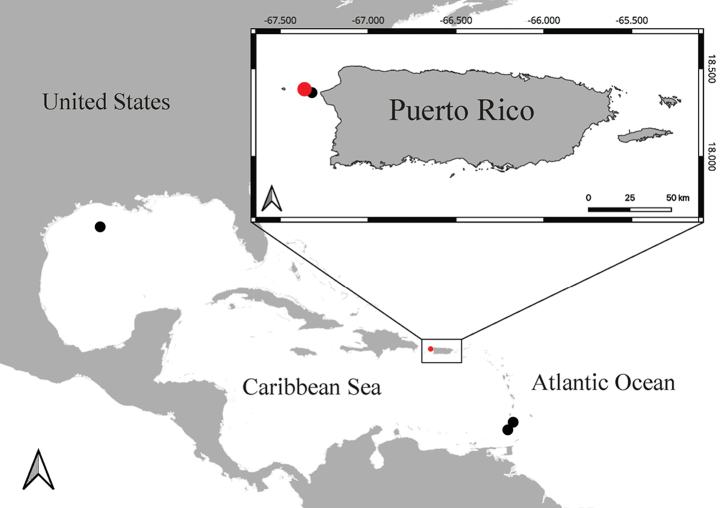
Locations where specimens of *Ameripathespseudomyriophylla* gen. nov., sp. nov. were collected (red dots indicate the type locality; black dots represent additional sample localities).

The specimen was collected 7 km east of Desecheo Island, Puerto Rico, in the Mona Passage, which connects the Atlantic Ocean to the Caribbean Sea, at a depth of 165 m during the Schmidt Ocean Institute expedition FKt230417 entitled: ‘Health diagnostics of deep-sea coral’ (Fig. 1) onboard the R/V Falkor (too). The colony was imaged with high-resolution video and sampled by cutting a 19 cm section of a branch using a manipulator arm of the ROV SuBastian. The holotype is this subsampled portion of the whole colony. One paratype was collected in the same way 6 km east of Desecheo Island. Both specimens are deposited in the collections of the National Museum of Natural History (**NMNH**), Smithsonian Institution, Washington DC.

The syntype of *Antipathespicea* was collected by the U.S. Coast Survey Steamer Blake in 1879 and is deposited in the collections of the Museum of Comparative Zoology at Harvard University (specimen registration prefix “MCZ:IZ:”). One of the remaining two paratypes was collected by the U.S. Bureau of Commercial Fisheries R/V Silver Bay in 1957 and the other by the R/V Pillsbury of the University of Miami in 1969, and both are deposited in the collections of the Rosenstiel School of Marine, Atmospheric and Earth Science at the University of Miami (specimen registration prefix “UMML”). The locations where the specimens were collected are indicated in Fig. 1.

### ﻿Morphological analyses

The skeletal elements of the specimens “new to science” were examined using a Zeiss EVO MA 15 scanning electron microscope (SEM). Before scanning, the specimens were coated with a 30–40 nm thick layer of 60% gold: 40% palladium. SEM stubs are deposited at the NMNH. SEM stub numbers are from a series established by the authors at the NMNH. The microscopic skeletal features were measured directly using a Meiji Techno RZ stereo microscope equipped with an ocular micrometer or from the photographs taken with the SEM. Spine height was measured as the distance from the spine tip to the middle of the base of the spine. Polyp and branch characteristics were measured with a dissecting microscope and terminal branch diameter was measured near the base of the branch. The morphological characters of the specimens were compared with all nominal and currently accepted families.

### ﻿Molecular analyses

DNA extractions of the holotype and one paratype were performed using the DNeasy Blood and Tissue Kit (Qiagen, Germany) following the manufacturer’s protocol. The DNA was cleaned with a Qiagen Power Clean Pro kit, and concentrations were estimated using a High Sensitivity Qubit 4 Fluorometer (Invitrogen, US). For the new species, DNA was sheared using a QSonica Inc Sonicator Q800R to a target size range of 400–800 bp and then checked via gel electrophoresis on a 1.5% agarose gel. Following shearing, DNA libraries were prepared with the Kappa Hyper Prep protocol using a ½ reaction with iTruSeq adapters and dual indexes following Quattrini et al. (2018). DNA extraction and library preparation were conducted in the Laboratories of Analytical Biology at the NMNH. Paired-end sequencing (150 bp) was performed on an Illumina - NovaSeq X Plus at the Oklahoma Medical Research Foundation Genomics Facility with other samples to obtain 10M paired-end (PE) reads (150 bp) per sample. Raw reads are deposited in the short read archive (SRA) of the National Center for Biotechnology Information (https://www.ncbi.nlm.nih.gov/).

### ﻿Phylogenetic analyses

The conserved loci were bioinformatically obtained from the high-throughput sequencing data. First, raw reads were trimmed using Trimmomatic v. 0.35 (Bolger et al. 2014) and then assembled using Spades v. 3.15 (Prjibelski et al. 2020). Then, UCE and exon loci were extracted using the hexacoral-v2-baitset (Cowman et al. 2020), following the Phyluce pipeline (https://phyluce.readthedocs.io/en/latest/tutorials/tutorial-1.html) (Faircloth 2016) with some modifications such as minimum-identity and minimum-coverage thresholds set to 70%. These data were then combined with existing conserved loci extracted from previous studies. All loci were edge-trimmed and aligned with MAFFT v. 7.130 (Katoh and Standley 2013). Then, phyluce_align_get_only_loci_ with_min_taxa was used to obtain all loci with 50% taxon-occupancy, which were then concatenated using phyluce_align_concatenate_alignments. The phylogenomic inference was conducted on the concatenated dataset using maximum likelihood analysis in IQTree v. 2.1 (Minh et al. 2020). A partitioned analysis (Chernomor et al. 2016) was conducted on the dataset using the best model for each locus [-m TESTMERGE (Kalyaanamoorthy et al. 2017)]. Ultrafast bootstrapping [-bb 1000 (Hoang et al. 2018)] and the Sh-like approximate likelihood ratio test [-alrt 1000 (Anisimova et al. 2011)] were also selected. All analyses were run on the Smithsonian’s High-Performance Computing Cluster (doi.org/10.25572/SIHPC), except for the phylogeny, which was plotted in FigTree v. 1.4.4.

## ﻿Taxonomic results

### ﻿Family Ameripathidae

Taxon classificationAnimaliaAntipathariaAmeripathidae

Opresko & Horowitz fam. nov.

DEDACB3A-28BA-5A96-84BC-52DCF820D885

https://zoobank.org/D9089DA2-C41B-414C-A224-D4DA55B133C7

#### Diagnosis.

Corallum branched and pinnulate; flabellate. Primary pinnules arranged bilaterally and alternately, secondary pinnules arranged uniserially. Spines up to 0.06 mm tall, conical, slightly compressed laterally with rounded apex, smooth or with knob-like protuberances. Polyps slightly transversely elongate up to 1.2 mm in transverse diameter, subequal tentacles, sagittals positioned lower than laterals, and a raised oral cone.

#### Remarks.

**Figure 2.**
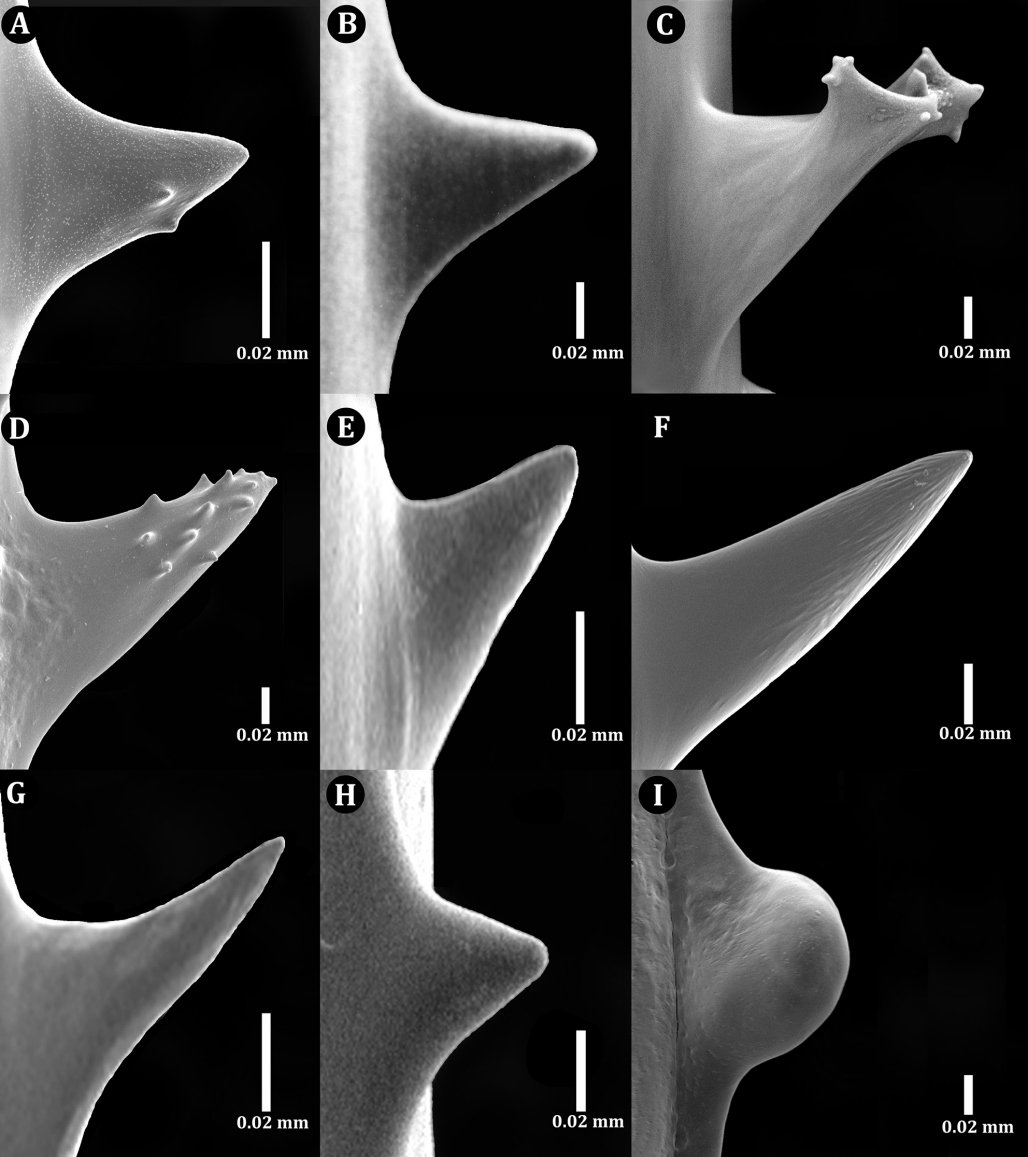
Skeletal spines of antipatharian families **A**Ameripathidae (*Ameripathespseudomyriophylla* gen. et sp. nov.) **B**Antipathidae (*Antipathesfurcata* Gray, 1857) **C**Antipathidae (*Antipathesfalkorae* Horowitz, 2022) **D**Aphanipathidae (*Aphanipathessarothamnoides* Brook, 1889) **E**Stylopathidae (*Stylopatheslitocrada* Opresko, 2006) **F**Myriopathidae (Myriopathescf.japonica (Brook, 1889)) **G**Cladopathidae (*Cladopathesplumosa* Brook, 1889) **H**Schizopathidae (*Schizopathesaffinis* Brook, 1889) **I**Leiopathidae (*Leiopathes* sp. of Hamie 1849).

Examination of SEM images of the spines of the new family revealed that most spines are triangular and smooth with un-ornamented surfaces with a small number of polypar spines possessing a few small, rounded, low-relief, knob-like protuberances (Fig. 2A). Also, the rows become much less regular; however, the number of rows per view, and the density of spines in each row do not differ substantially from that on the pinnules (Fig. 6D–G). These spine characteristics differ from other families, for example: Antipathidae Ehrenberg, 1834 has spines that are either perfectly smooth (Fig. 2B) or are papillose with or without apical bifurcations or multiple knobs (Fig. 2C). Aphanipathidae Opresko, 2004 typically has spines with distinct tubercles (Fig. 2D). Stylopathidae Opresko, 2006 has spines that are small, conical, smooth-surfaced, and distally directed (Fig. 2E). Myriopathidae Opresko, 2001 has blade-like spines, often with very small, elongated papillae or fine striations (Fig. 2F), and in which the spines increased in density on the larger branches and stem. Cladopathidae Kinoshita, 1910 has spines that are smooth, triangular, or conical, and often distally directed (Fig. 2G). Schizopathidae Brook, 1889 has spines that are simple or multi-lobed, smooth, and conical or triangular (Fig. 2H). Leiopathidae Haime, 1849 has small spines that are simple or multi-lobed, smooth and are triangular, conical, or blister-shaped (Fig. 2I); often poorly developed or absent on older parts of the corallum (Molodtsova 2011).

**Figure 3.**
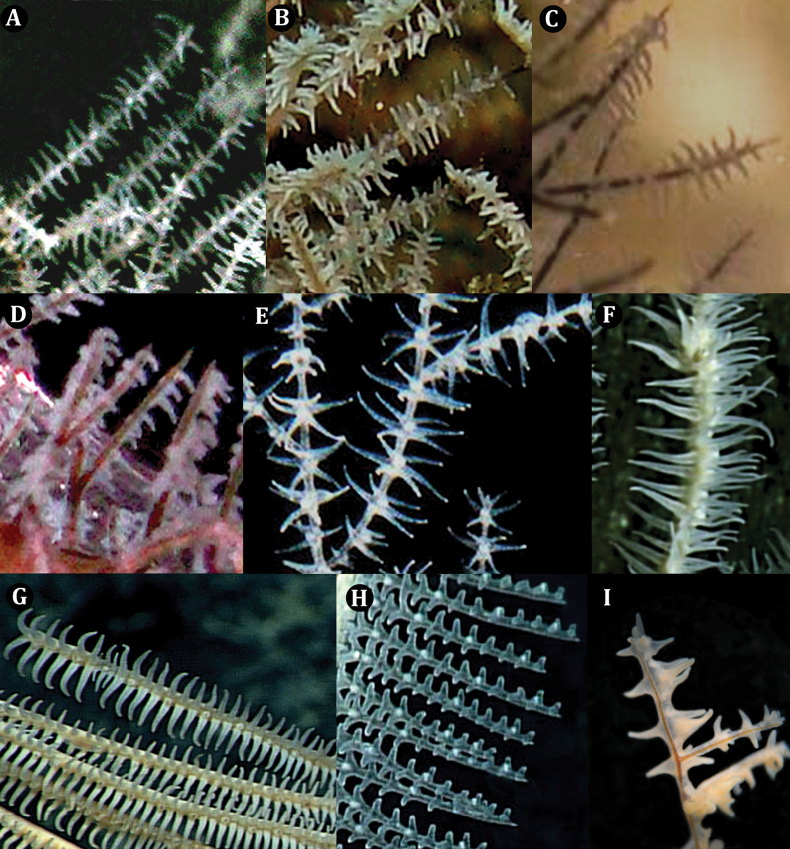
Polyps of live corals representing different antipatharian families **A**Ameripathidae (*Ameripathespseudomyriophylla* gen. et sp. nov.) **B**Myriopathidae (*Myriopathes* sp.) **C**Stylopathidae (*Stylopathes* sp.) **D**Stylopathidae (*Stylopathes* sp.) **E**Antipathidae (*Antipathesatlantica* Gray, 1857) **F**Aphanipathidae (*Anozopathes* sp. Opresko & Bo, 2021) **G**Schizopathidae (*Bathypathes* sp.) **H**Cladopathidae (*Heteropathes* sp.) **I**Leiopathidae (*Leiopathes* sp.). Photo credits: **A, C, D** (Schmidt Ocean Institute); **B** (M. Bo); **E** (S. Weinberg); **F, G, H** (NOAA/OER; **I** (P. Etnoyer).

Examination of in situ images of the polyps of the new family revealed a slightly transversely elongated external morphology (Fig. 3A). The tentacles are subequal in length, cylindrical with rounded tips and are relatively short, being no more than 1.5× the transverse diameter of the polyp. The number of mesenteries in the polyps of the Ameripathidae has not yet been determined. The new family has polyps like Myriopathidae (Fig. 3B); except in Myriopathidae, polyps are more circular in shape with tentacles equally distributed and positioned around the polyp mouth. The new family also has polyps like Stylopathidae (Fig. 3C, D); except polyps are not as transversely elongated as the new family. The remaining families have different polyp characteristics: The polyps of the Antipathidae (Fig. 3E) and Aphanipathidae (Fig. 3F) tend to be as long as wide (sometimes transversely compressed), and with expanded tentacles ≤ 3× the transverse diameter of the polyps and tentacles are not subequal in length. The polyps of Schizopathidae (Fig. 3G) and Cladopathidae (Fig. 3H) are more transversely elongated than the new family. Leiopathidae polyps are transversely compressed (Fig. 3I).

#### Comparative diagnosis

Antipathidae differs in corallum pattern with an unpinnulated corallum compared to the pinnulated corallum in the new family. Cladopathidae differs in polyp size and spine ornamentation, with polyps measuring at least 1.8–2.0 mm compared to up to 1.2 mm in transverse diameter in the new family and possessing smooth spines rather than ones with knob-like protuberances. Leiopathidae differs in corallum pattern, possessing an unpinnulated corallum compared to a pinnulated corallum in the new family. Aphanipathidae differs in spine height, spine ornamentation type, and polyp characteristics, possessing spines up to 0.5 mm tall, distinct tubercles on the surfaces of skeletal spines, and polyps that are as long as they are wide or can be transversely compressed, respectively, compared to spine heights 0.06 mm tall, low-relief, knob-like protuberances on the surfaces of skeletal spines, and slightly transversely elongate polyps with short, subequal tentacles with blunt, rounded tips in the new family. Myriopathidae differs in spine ornamentation type with striations on the surfaces of skeletal spines compared to the spines with a few small, rounded, low-relief, knob-like protuberances in the new family. Stylopathidae differs in subpinnule arrangement and spine surface ornamentation, presenting verticils or irregularly bilateral and smooth spines, respectively, compared to uniserially arranged subpinnules and spines with low-relief, knob-like protuberances spines in the new family. Schizopathidae differs in polyp size and spine ornamentation, having larger polyps (2–12 mm in transverse diameter) and smooth spines, respectively, compared to smaller polyps (up to 1.2 mm in transverse diameter) and spines with knob-like protuberances in the new family.

**Figure 4.**
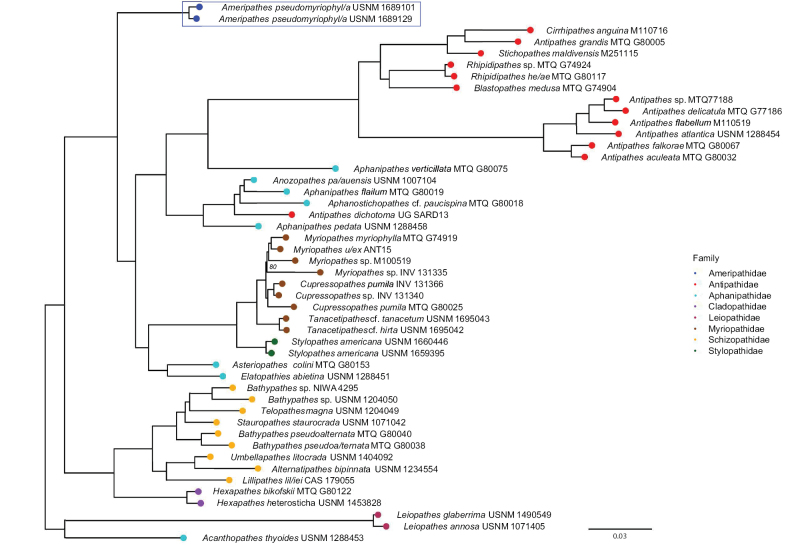
Maximum likelihood phylogeny of all black coral families and the new family based on a 50% complete matrix containing 741 loci. Boxed taxa (blue outline) represent the new species. Ultrafast bootstrap support is 100% at all nodes except for two nodes, which are noted in the phylogeny. The phylogeny was rooted to the Leiopathidae clade based on results from Horowitz et al. (2023b).

The recognition of the new family is further supported by the phylogenetic analysis (see further below) conducted on the holotype and one paratype showing that Ameripathidae is a distinct lineage sister to the Antipathidae + Aphanipathidae + Myriopathidae + Stylopathidae, representing a novel deep divergence in the order Antipatharia (Fig. 4).

### ﻿Genus Ameripathes

Taxon classificationAnimaliaAntipathariaAmeripathidae

Opresko & Horowitz gen. nov.

7032E12E-B887-558A-9C71-E9048E8C137F

https://zoobank.org/18F1C58B-E0CE-462A-A160-4497CCC150BF

#### Type species.

*Ameripathespseudomyriophylla* sp. nov. (see below).

#### Diagnosis.

Corallum sparsely branched, generally in one plane to the seventh order or more. Stem and branches consistently pinnulated to the second order and very rarely to the third order on older sections of the corallum, in some specimens. Primary pinnules 1–2 cm in length, thin; < 0.1 mm in diameter at their midsection. Primary pinnules arranged bilaterally and alternating in two rows. Secondary pinnules 0.5–2 cm in length; arranged uniserially at intervals starting near the base of primary pinnules, projecting anteriorly. Rarely, one or two tertiary pinnules occurring on a very small number of secondary pinnules and usually on the most basal secondary pinnules on the older portions of the corallum. Spines conical, slightly compressed laterally; ≤ 0.06 mm tall on the pinnules; mostly smooth, but some polypar spines possessing small, rounded, low-relief, knob-like protuberances. Spines taller, ≥ 0.14 mm, and needle-like on the thicker branches and stem. Number of spine rows per view and the density of spines in each row do not differ substantially from that on the pinnules. Polyps up to 1.25 mm in transverse diameter, appearing elongated transversely, arranged in a single row, with mostly seven or eight polyps per centimeter. Tentacles cylindrical, subequal in size; and when fully extended not much longer than the transverse diameter of the polyps. Tip of expanded tentacles rounded. Oral cone raised.

#### Remarks.

Although the family at present contains only a single genus, a comparison with genera of other families suggests that the major generic morphological feature of *Ameripathes* is the pinnulate nature of the corallum, with two bilateral rows of primary pinnules and one to two orders of subpinnules. This same pinnulation pattern occurs in the genus *Myriopathes* in the family Myriopathidae and in the deep-sea genus *Dendrobathypathes* Opresko, 2002 in the family Schizopathidae. The occurrence of similar branching or pinnulation patterns across families is common among antipatharians.

Another feature that occurs in both *Ameripathes* and *Myriopathes* is an increase in the size of the spines on the branches and stem where they are more cylindrical and needle-like. However, in *Ameripathes* the spines on the stem do not increase in density (number of rows and number per row) and do not become forked or antler-shaped, which is often the case in the *Myriopathes*.

#### Species assigned to *Ameripathes*.

*Ameripathespseudomyriophylla* sp. nov. is the only species assigned to the newly described genus.

#### Etymology.

In accordance with Article 11.3 of the International Code for Zoological Nomenclature the name of the genus *Ameripathes* can be considered an arbitrary combination of letters; however, it is loosely derived from “americana” in reference to the early association of this taxon with *Antipathesamericana* Duchassaing and Michellotti, and the commonly used suffix -*pathes*.

#### Distribution.

The single species assigned to this genus is known only from the Western Central Atlantic, in the Caribbean Sea and the Gulf of Mexico (Fig. 1).

### ﻿ Ameripathes pseudomyriophylla

Taxon classificationAnimaliaAntipathariaAmeripathidae

Opresko & Horowitz sp. nov.

615AE736-1C23-5A91-B4E3-985420EA97D8

https://zoobank.org/9D4AFE4A-C83F-49A7-81BE-8E45999B0B56

**Figure 5.**
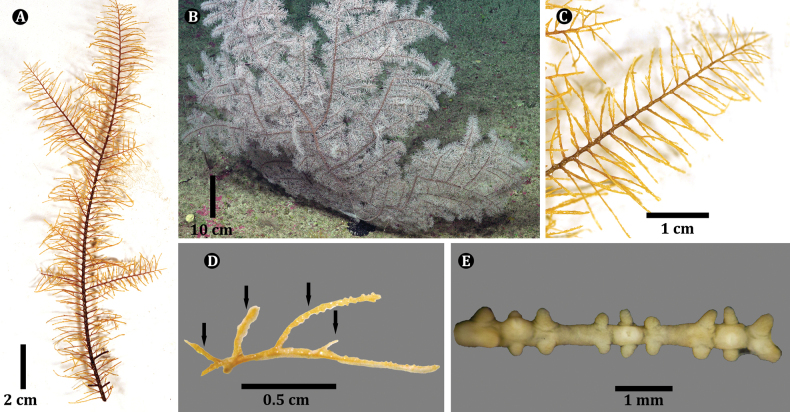
*Ameripathespseudomyriophylla*, holotype (USNM 1689129) **A** section of the collected specimen **B** colony from which the holotype specimen was collected **C** section of pinnulated branch showing two rows of alternating primary pinnules and uniserial secondary pinnules **D** dorsal view of pinnulation pattern (second-order uniserial pinnules indicated by the arrows) **E** section of pinnule showing preserved polyps with contracted tentacles.

**Figure 6.**
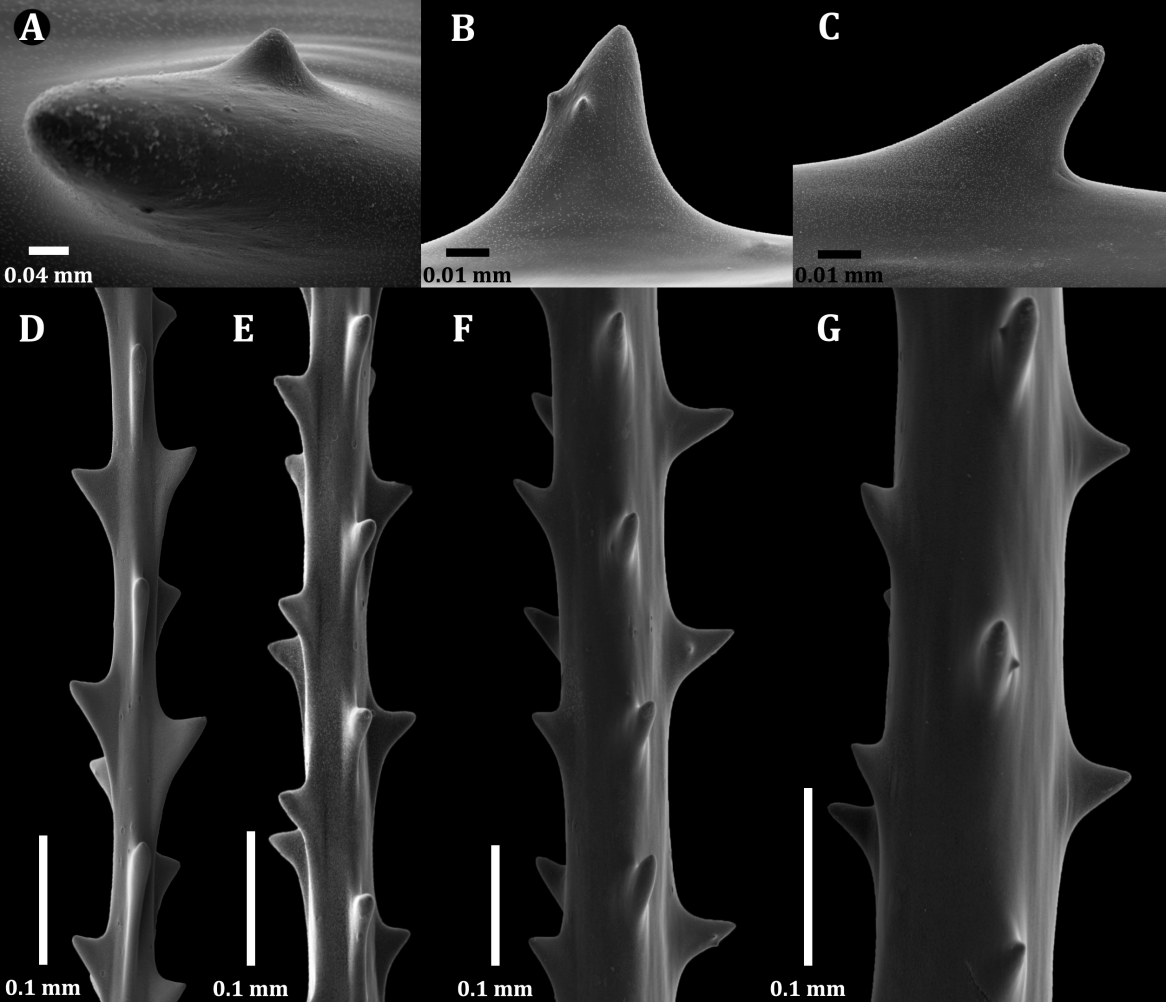
*Ameripathespseudomyriophylla*, holotype (USNM 1689129) **A** dorsal view of single spine showing a single knob **B** lateral view of a polypar spine showing two knobs **C** lateral view of an abpolypar spine **D–G** sections of pinnules of increasing thickness.

**Figure 7.**
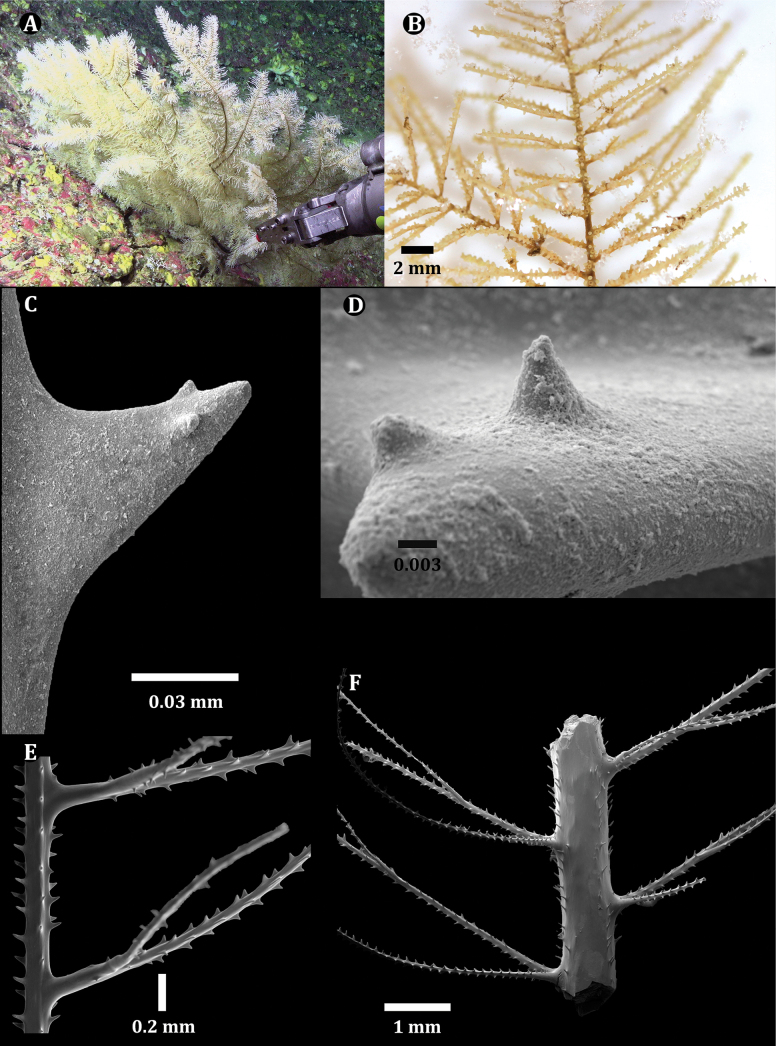
*Ameripathespseudomyriophylla*, paratype (USNM 1689101) **A** colony from which the paratype was collected **B** section of the collected specimen **C** lateral view of a polypar spine showing two knobs **D** dorsal view of single spine showing two knobs **E** lateral view of a pinnulated branch showing the arrangement of the pinnules and subpinnules **F** lateral view of a branch showing the arrangement of the pinnules and subpinnules.

**Figure 8.**
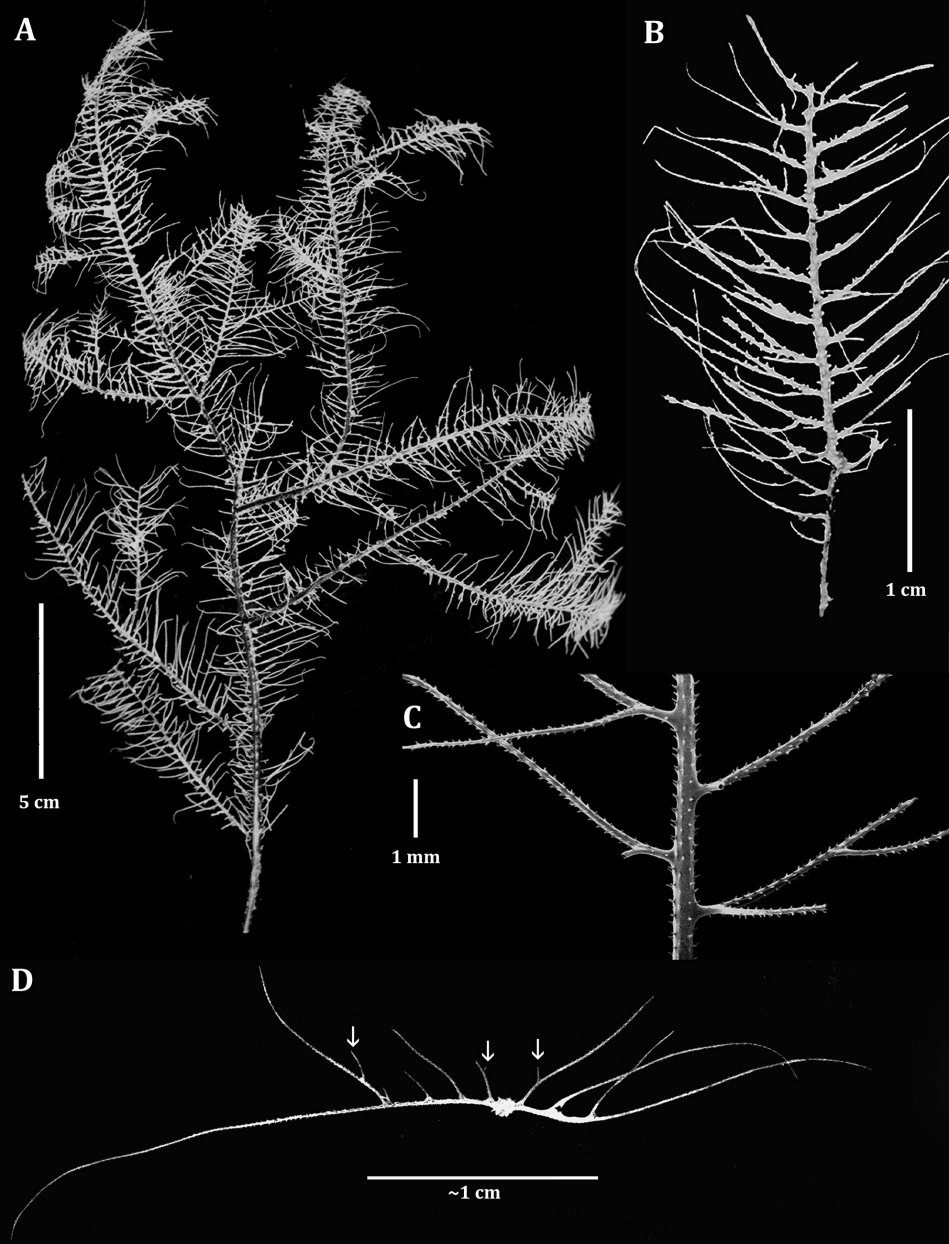
*Ameripathespseudomyriophylla*, paratype (UMML 7.669) **A** entire corallum **B** section of a branch **C** closeup view showing the arrangement of the pinnules and subpinnules **D** dorsal view of pinnulation pattern (third order pinnules indicated by the arrows).

**Figure 9.**
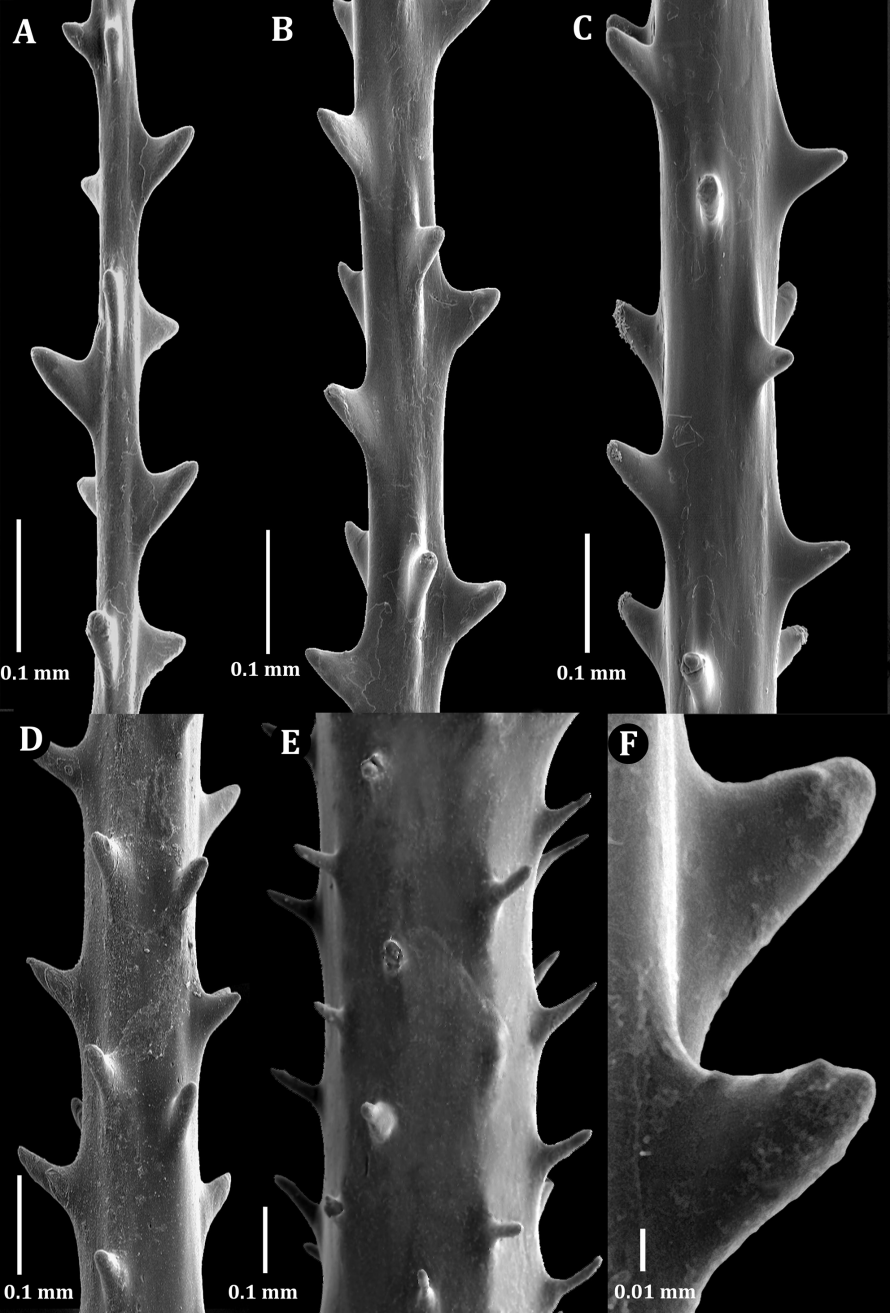
*Ameripathespseudomyriophylla*, paratype (USNM 1705331, subsample of UMML 7.669) **A–C** sections of pinnules **D** branchlet **E** section of thick branch **F** close-up view of two spines showing knob-like protuberances.

Figs 2A , 3A , 5 , 6 , 7 , 8 , 9 , Suppl. material 1

Antipathes americana Opresko, 1972: 975–979.
Antipathes picea Pourtalès, 1880: 115 (in part).
not Antipathesamericana Duchassaing & Michelotti, 1860: 56.

#### Material examined.

***Holotype***: USNM 1689129, 7 km east of Desecheo Island, Puerto Rico, 18.387°N, 67.408°W, 165 m depth, seawater temperature 22 °C. Schmidt Ocean Institute R/V Falkor (too), FKt230417, *Health diagnostic of deep-sea coral*, ROV SuBastian dive 506, April 19, 2023 (SEM stub No. 586–590). ***Paratypes***: USNM 1689101, 6 km east of Desecheo Island, Puerto Rico, 18.389°N, 67.417°W, 183 m depth, seawater temperature 21 °C. Schmidt Ocean Institute R/V Falkor (too), FKt230417, *Health diagnostic of deep-sea coral*, ROV SuBastian dive 504 April 18, 2023 (SEM stub No. 581–585); UMML 7.669 (schizoparatype USNM 1705331), Lesser Antilles, off Carriacou, 12.3917°N, 61.3600°W, 37–251 m depth. R/V *Pillsbury* sta. 857, July 3, 1969 (SEM stub No. 223); UMML 7.668 (schizoparatype USNM 1705332), Gulf of Mexico, 28.1167°N, 95.05°W, 55 m depth, R/V Silver Bay sta 190, Sept. 27, 1957; MCZ:IZ:57354, Lesser Antilles, off Grenada, 12.0583°N, 61.7861°W, 532 m depth, USCSS Blake sta. 260, Feb. 28, 1879 (SEM stub No. 207) (syntype of *Antipathespicea* Pourtalès).

#### Type locality.

7 km east of Desecheo Island, Mona Passage, Puerto Rico, 165 m depth

#### Diagnosis.

As for the genus.

#### Description of the holotype.

The holotype (USNM 1689129) is a 19-cm section of branch (Fig. 5A) from a colony estimated to be ~ 0.75 m tall based on in situ imagery (Fig. 5B). The polyps are white in color, both in situ and in the preserved state. The collected sample is sparsely branched to the second order, and pinnulate to the second order. Pinnulate branches are 4–6 cm in length and occur irregularly, but bilaterally. The primary pinnules, arranged bilaterally and alternately along the branches (Fig. 5C), measure 1–2 cm in length with basal diameters ≤ 0.2 mm, tapering to ~ 0.05 mm midsection and 0.02 mm near the distal end, are spaced 2.0–2.5 mm apart in each row (8 or 9 per centimeter, total for both rows), and incline distally at ~ 75^0^. The interior angle between the two rows of primaries is ~ 150^0^. The pinnules also tend to curve posteriorly, towards the abpolypar side of the corallum. The secondary pinnules are arranged uniserially on the polypar side of the primary pinnules; the most proximal secondary is placed ~ 2 mm from the base of the primary pinnule, and secondary pinnules are spaced between 2–5 mm apart in a row with a maximum of three secondary pinnules occurring on one primary pinnule (Fig. 5D). The secondary pinnules are mostly 0.5–2.0 cm long and extending out from the polypar side of the corallum and form 60° distal angles. The longest secondaries can be longer than the primary pinnule from which they arise.

Spines (Fig. 6) on the pinnules are conical, slightly compressed laterally (especially those nearer the tips of the pinnules), with a rounded apex, and smooth or with one to three small, rounded, low-relief, knob-like protuberances 0.003–0.006 mm tall on polypar spines visible in lateral view (Fig. 6A, B). On pinnules ranging between 0.03 and 0.1 mm in diameter, polypar spines are 0.05 to 0.06 mm in height (Fig. 6B), and abpolypar spines range from 0.04 to 0.05 mm (Fig. 6C). The abpolypar spines are more distally directed than polypar spines. The developing spines nearer to the tip of the pinnules, where the axial diameter is 0.02–0.04 mm, have a very elongated and sloping proximal edge, ≤ 0.1 mm long, and a very short distal edge of ~ 0.03 mm or less, which extends out at near right angles to the surface of the pinnule (Fig. 6D). The distance from the tip of the spines to the middle of the base; however, is 0.05–0.06 mm. Three to four rows of spines can be counted in one view. The spines in each row are offset such that they also appear to follow a spiral pattern around the axis (with one spine from each row). Spaces between spines in a row range from 0.18 mm (abpolypar spines) to 0.22 mm (polypar spines), and 6 spines can be counted in 1 mm in each row. Spines on branches become taller and more cylindrical and needle-like; on a branch 1.3 mm in diameter, they are ≤ 0.12 mm tall. The rows become much less regular; however, the number of rows per view, and the density of spines in each row do not differ substantially from that on the pinnules.

Polyps occur in a single row. On the primary pinnules they are confined on or near the side on which the subpinnules occur. The polyps are 1.0–1.25 mm in the transverse diameter (Fig. 5E). They appear elongated due to the small axial diameter of the pinnules. The interpolypar distance (between lateral tentacles of adjacent polyps) is 0.4–0.6 mm, and there are usually seven or eight polyps per centimeter. The tentacles (in preserved state) are subequal in size, 0.4–0.5 mm long, with the sagittals placed lower than the laterals. The oral cone is raised ~ 0.25 mm (Fig. 5E).

#### Description of colony from which holotype was collected.

The colony from which the holotype was collected was imaged in situ (Fig. 5B) and based on a 4K video (https://tinyurl.com/SupplMat2), the complete colony was estimated to be approximately 1 m tall and 0.75 m wide. The main stem is ~ 1 m tall and the colony is branched to the seventh order or more and forms a distinct fan shape. Branches are ≥ 20 cm in length, have 75–90^0^ distal angles, and are straight or curved distally near the base of the lower order branch from which they arise, and are slightly curved proximally towards the tip. Branches occur on both sides of lower-order branches.

#### Description of paratypes.

Specimen USNM 11689101 is a 12-cm section of branch from a colony estimated to be ~ 0.3 m tall based on in situ imagery (Fig. 7A). The polyps are white in color in situ and in the preserved state. The collected sample has two orders of branches and pinnulate to the second order. The primary pinnules, arranged bilaterally and alternately along the stem and branches (Fig. 7B), measure 1.0–1.5 cm in length with basal diameters ≤ 0.1 mm, tapering to ~ 0.05 mm midsection and 0.03 mm near the distal end, are spaced 2.0 mm apart in each row (9 or 10 per centimeter, total for both rows), and incline distally at ~ 65^0^. The interior angle between the two rows of primaries is ~ 130^0^. The pinnules also tend to curve posteriorly, towards the abpolypar side of the corallum. The secondary pinnules are arranged uniserially on the polypar side of the primary pinnules; the most proximal secondary is placed ~ 2 mm from the base of the primary pinnule and rarely < 1 mm from the base of the primary pinnule. Secondary pinnules are spaced between 2–5 mm apart in a row with a maximum of two secondary pinnules occurring on one primary pinnule (Fig. 7B). The secondary pinnules are mostly 0.5–1.5 cm long and extending out from the polypar side of the corallum and form 60^0^ distal angles. The longest secondaries can be longer than the primary pinnule from which they arise.

Spines on the pinnules are conical, slightly compressed laterally (especially those nearer the tips of the pinnules), with a rounded apex, and smooth or with one to two small, rounded, low-relief, knob-like protuberances 0.003–0.006 mm tall on polypar spines visible in lateral view (Fig. 7C). On pinnules ranging between 0.03 and 0.08 mm in diameter, polypar spines are 0.04–0.07 mm in height, and abpolypar spines range from 0.04 to 0.05 mm. The abpolypar spines are more distally directed than polypar spines. Three rows of spines can be counted in one view. The spines in each row are offset such that they also appear to follow a spiral pattern around the axis (with one spine from each row). Spaces between spines in a row range from 0.2 to 0.23 mm among abpolypar spines and to 0.25–0.3 among polypar spines, and five or six spines can be counted in 1 mm in each row. Spines on branches become taller and more cylindrical and needle-like and less regularly arranged, and sometimes absent on large areas of the axial surface (Fig. 7D). On a branch 0.8 mm in diameter, the spines are ≤ 0.13 mm tall, in four irregular rows and with mostly five or six spines per millimeter in each row (Fig. 7D).

Polyps occur in a single row. On the primary pinnules they are confined on or near the side on which the subpinnules occur. The polyps are 1.0–1.25 mm in the transverse diameter (Fig. 7D). They appear elongated due to the small axial diameter of the pinnules. The interpolypar distance (between lateral tentacles of adjacent polyps) is 0.1–0.2 mm and there are usually eight or nine polyps per centimeter. The tentacles (in preserved state) are subequal in size with the sagittals placed lower than the laterals. The oral cone is raised ~ 0.25 mm.

The specimen from R/V Pillsbury sta. 857 (UMML 7.669, Fig. 8A) is mostly flattened in one plane, but with some overlapping branches. It has a height of 28 cm, a width of 22 cm, and a basal stem diameter of ~ 2 mm. It is sparsely branched to the third order, and pinnulate to the second and occasionally third order. The major branches are 5–10 cm in length and spaced 2.5–3 cm apart, and extend out laterally to vertically. As in the holotype, the primary pinnules are arranged bilaterally and alternately along the stem and branches (Fig. 8B, C). They are 1.5–2.0 cm long, mostly around 0.06 mm thick (up to 0.1 mm near the base), 2.5–3.0 mm apart (eight or nine per centimeter, total for both rows), and most are inclined distally, usually forming an angle of ~ 60^0^ with the branch from which they arise. The interior angle between the two rows of primaries is close to 180^0^. The pinnules also tend to curve posteriorly, towards the abpolypar side of the corallum. The secondary pinnules are arranged uniserially on the polypar side of the primary pinnules (Fig. 8C); the lowermost secondary is placed ~ 0.5 mm from the base of the primary, the second is 1.2–2.6 mm from the lowermost one, and the third secondary is 1.7–2.8 mm from the second. There can be as many as four secondary pinnules on the longest primaries. The secondary pinnules are 0.5–2.0 cm long and are inclined distally such that they form a distal angle of ~ 60^0^ with the primary pinnule. The longest secondaries are usually those nearest the base of the primary, and they are sometimes as long, or longer than the primary pinnule from which they arise. One or two tertiary pinnules are rarely present on the polypar side of the lowermost secondary pinnules and sometimes on one of the more distal secondaries (Fig. 8D).

Spines (Fig. 9) on the pinnules are conical, slightly compressed laterally (especially those nearer the tips of the pinnules), with a rounded apex, and smooth or with a few small, rounded, low-relief, knob-like protuberances. The spines on one side are more distally directed than those on the opposite side. On the distal portion of a pinnule, where the axial diameter ranges from ~ 0.03 to 0.06 mm, the polypar spines are mostly 0.05–0.06 mm tall, and the abpolypar spines ~ 0.04 mm (Fig. 9A, B). On a pinnule with an axial diameter of 0.08–0.1 mm, the spines are ~ 0.06 mm (Fig. 9C–F). Spines on branches and stem become taller and more cylindrical and needle-like; on a branch 0.3 mm in diameter, they are ≤ 0.11 mm tall, spaced 0.2–0.3 mm apart (~ 5 per millimeter) and arranged in five rows as seen from one aspect (Fig. 9E).

Polyps occur in a single row. On the primary pinnules they are confined on or near the side on which the subpinnules occur. The polyps are 0.6 mm in transverse diameter near the tips of the pinnules, increasing to ~ 1.1 mm near the base. They appear elongated due to the small axial diameter of the pinnules. The interpolypar distance (between lateral tentacles of adjacent polyps) is 0.1–0.2 mm, and there are usually eight or nine polyps per centimeter, rarely as many as ten per centimeter. The tentacles (in preserved state) are subequal in size, ~ 0.4 mm long, with the sagittals placed lower than the laterals. The oral cone is ~ 0.25 mm high.

The MCZ specimen from *Blake* Sta. 260 is a 6.5-cm long branch or the stem of a small colony (with the basal plate missing). The primary pinnules are 1–2 cm long, ~ 0.17 mm in diameter at their base, and spaced ~ 3.0 mm apart in each row. Primary pinnules longer than ~ 2 cm develop into pinnulated branches with the primary pinnules 3 mm apart in each lateral row. The interior angle formed by the two rows of primary pinnules is 160–175^0^, but the pinnules are curved backwards so that they appear in the plane of the stem/branch. The distal angle of the pinnules is ~ 75^0^. There are ≤ 4 secondary pinnules on each primary; the first is 0.05 mm from the base of the primary, the second 1.5–1.8 mm from the first, the third 2.0–2.5 mm from the second; and the fourth 3 mm from the third. The secondary pinnules are 0.2–0.7 cm long. They extend outward on the convex side of the primaries and are also angled distally ~ 60^0^ to the primary. Tertiary pinnules are present on a few of the basal-most secondaries and are in the same plane as the secondary or extend upward to be parallel to the branch. The spines are 0.06 mm tall on the pinnules and 2–3× taller on the lower part of the stem/branch (≤ 0.17 mm). They are less regularly arranged near the base of the stem and extend out in various directions. In places, they are completely absent. Polyps are not present.

The specimen from Silver Bay sta. 190 (UMML 7.668) is 15 cm high and 8 cm wide, with a stem ~ 2.5 mm in diameter near its basal end. In this colony, the secondary pinnules are 0.5–1.5 cm long, and tertiary pinnules occur on not only the most basal secondary pinnule but also on the more distal ones. The tertiary pinnules also tend to project upward, parallel to the branch. On the lower part of the stem, the skeletal material of the axis has overgrown the basal portion of the lowermost secondary pinnules. Consequently, the stem appears to have four rows of primary pinnules. This appearance is enhanced by the fact that the secondary pinnules can be longer than the primary pinnules.

#### Intraspecies variation.

The five specimens in the type series are fairly consistent in morphological features. They all form a branched pinnulate corallum with at least two orders of pinnules. The colonies tend to be planar. The occurrence and number of secondary and tertiary pinnules is, however, variable, both within a colony and between colonies, explainable perhaps by the size and age of the colony or the section of the colony sampled. The primary pinnules are always arranged bilaterally and alternately in two rows. The distance between primary pinnules in each row ranges from 2 to 3 mm; the total density for both rows is fairly consistent at eight or nine per centimeter. On the largest specimens, the maximum length of the primary pinnules is 2 cm or slightly longer. Pinnules longer than 2 cm usually develop into pinnulated branches. The pinnules are always very thin, the axial diameter is usually < 0.2 mm near their insertion on a branch and only 0.05–0.06 mm at their midpoint. The number of secondary pinnules per primary pinnule is usually one or two; however, rarely, there are as many as four per primary. Secondary pinnules are variable in length within and between colonies and can be as long as the primary pinnules. Tertiary pinnules are very rare and appear to occur mainly on older sections of the corallum. The fact that tertiary pinnules could not be found on the holotype is most likely due to its being a section taken from the upper, younger part of the colony. When present, tertiary pinnules are very short and are mostly confined to the lowermost secondary pinnules; however, they occasionally can also be found on a more distal secondary pinnule. There are usually only one, and rarely two tertiary pinnules on a secondary. On the pinnules, the maximum size of the polypar spines is consistently 0.06 mm from colony to colony. The spines always increase in size and become more cylindrical and needle-like on the larger branches, and depending on the axial diameter, can be as tall as 0.17 mm. The number of rows of spines varies slightly from colony to colony ranging between three or four to four or five visible in one aspect.

The number of rows, however, does not increase on the larger branches, and the density of the spines is mostly five or six per mm, even on the thickest branches. The maximum size of the polyps varies only slightly between colonies in that the transverse diameter ranges from ~ 1–1.25 mm, and the density is typically 7–9 per cm. The interpolypar distance ranges from 0.1 to 0.6 mm.

#### Phylogenetic results.

A total of 60–986 conserved element loci were obtained per specimen. Total number of contigs ranged from 16,819 to 1,444,028 base pairs (bp) (average lengths (bp) ranged from 280 to 1,695). The 50% taxon occupancy matrix included 741 loci that were concatenated into an alignment with a total length of 391,648 bp. Read and locus summary statistics are detailed in Suppl. material 1. The maximum likelihood phylogeny includes all eight black coral families with the new family representing a distinct lineage sister to the Antipathidae + Aphanipathidae + Myriopathidae + Stylopathidae (Fig. 4).

#### Etymology.

The species name is derived from *pseudo* (false) and *myriophylla*, in reference to the very similar appearance to species in the genus *Myriopathes* with *M.myriophylla* being the type species of the genus.

#### Distribution.

The species is only known only from the Caribbean and the Gulf of Mexico between 54 and 532 m depth.

## ﻿Discussion and conclusions

This study describes the first new black coral family discovered in 18 years, underscoring the untapped potential for groundbreaking discoveries even in seemingly well-surveyed areas, such as the Gulf of Mexico. Furthermore, the identification of this new species, alongside another recent species described from the Caribbean Sea off Puerto Rico (Horowitz et al. 2023a), further demonstrates the need to increase exploration efforts of mesophotic and deep coral habitats, particularly in understudied regions. Discoveries such as this are now more feasible than ever, thanks to sophisticated remotely operated vehicles (ROVs) that can collect coral species with precision and provide invaluable in situ data, including imagery that reveals information such as colony color, habitat, and associated invertebrates. Furthermore, the advent of cutting-edge genetic methodologies for black corals–specifically, genome skimming and target enrichment of conserved elements (see Quattrini et al. 2018, 2024)–has revolutionized our ability to place these specimens within a phylogenetic framework with unparalleled accuracy and resolution, thereby bolstering the foundation for erecting new taxa.

Traditionally, the taxonomic classification of black corals has relied heavily on morphological characters, which can be prone to issues of convergence and homoplasy (Horowitz et al. 2023a). This study, however, leverages robust molecular data to navigate the complexities of classification, especially when the new species shares branching, spine, and polyp characteristics with the families Myriopathidae and Stylopathidae. By integrating detailed morphological examinations with advanced molecular analyses, this research confidently positions the new species within a novel genus and family and reinforces the importance of an integrated taxonomic framework. Such research not only contributes to the scientific endeavor of cataloging earth’s biodiversity but also has profound implications for conservation strategies, offering insights that are vital for the preservation of vulnerable and often endangered ecosystems.

## Supplementary Material

XML Treatment for Ameripathidae

XML Treatment for Ameripathes

XML Treatment for Ameripathes pseudomyriophylla

## ﻿Additional information

### Author ORCIDs

Jeremy Horowitz https://orcid.org/0000-0002-2643-5200

Dennis M. Opresko https://orcid.org/0000-0001-9946-1533

Santiago Herrera https://orcid.org/0000-0001-7204-9434

Colleen M. Hansel https://orcid.org/0000-0002-3506-7710

Andrea M. Quattrini https://orcid.org/0000-0002-4247-3055

### Data availability

Raw sequence reads were submitted to GenBank under BioProject # PRJNA1078781. 4K video can be found on https://tinyurl.com/SupplMat2.

## Supplementary materials

Supplementary material 1 Table of read and locus summary statistics for specimens https://doi.org/10.3897/zookeys.1203.121411.suppl1 Data type xlsx Explanation note Metadata for molecular data including the number of raw reads, assembly statistics, and NCBI details. https://binary.pensoft.net/file/1060135This dataset is made available under the Open Database License (http://opendatacommons.org/licenses/odbl/1.0/). The Open Database License (ODbL) is a license agreement intended to allow users to freely share, modify, and use this Dataset while maintaining this same freedom for others, provided that the original source and author(s) are credited.Jeremy Horowitz, Dennis M. Opresko, Santiago Herrera, Colleen M. Hansel, Andrea M. Quattrini
